# Optimizing the Role of Registered Practical Nurses in the Operating Room: A Two-Phase Qualitative Descriptive Study

**DOI:** 10.1177/08445621251345337

**Published:** 2025-06-03

**Authors:** Sherry Espin, Sue Bookey-Bassett, Alyssa Indar, Victoria Pringle, Don Rose, Elaine Santa Mina, Juliette Teodoro

**Affiliations:** 1 7984 Toronto Metropolitan University, Daphne Cockwell School of Nursing, Toronto, ON, Canada; 2 7989 University Health Network, Collaborative Academic Practice, Toronto, ON, Canada

**Keywords:** Registered practical nurses, role utilization, operating room, health human resource planning, scope of practice

## Abstract

**Background:**

Current nursing shortages are shifting approaches to health human resource planning. Broad changes are being implemented to support system planning, however, there is a need to engage in targeted strategies that address shortages in specialty nursing areas, such as the operating room.

**Purpose:**

The purpose of this study was to explore how Registered Practical Nurses (RPNs) are currently utilized within operating room settings in Ontario, Canada.

**Methods:**

A two-phase qualitative descriptive study design was conducted. Phase 1 consisted of an online survey and Phase 2 consisted of individual, semi-structured virtual interviews. Participants included nurses working in urban and community hospitals and/or private clinics. Descriptive statistics were used to report participant demographic data, and qualitative data were analyzed using inductive content analysis.

**Results:**

Sixty-five participants completed the survey, and 13 participants completed the semi-structured interviews. Participants identified differences in RPN role utilization within different healthcare settings, teamwork and work culture. Recommendations for RPN leadership opportunities, policy support, professional development, and the role of professional nursing organizations were also identified.

**Conclusion:**

Given the complex nature of healthcare systems, new models of care, and evolving scopes of practice for healthcare providers, it is important to consider how RPNs can be further utilized to support patient care including specialty areas. Re-evaluating the roles and responsibilities of RPNs in healthcare is essential to strengthen the nursing workforce and prepare for ongoing human resource challenges.

## Background and Purpose

Current global nursing shortages are shifting approaches to health human resource planning (Sutton et al., 2023). In addition to broad changes that support whole-system planning, there is a need to engage in targeted strategies that address critical shortages in specialty nursing areas, such as the operating room (Ball et al., 2015; Brand et al., 2017). The shortage of perioperative nurses has been linked to longer surgical wait times, an important health quality system indicator (Rozario et al., 2024). Current strategies to address perioperative nursing recruitment and retention include enhancing education, improving academic/health system partnerships to develop a pathway to employment, and exploring alternate models of care to maximize nursing scopes of practice (Brooks et al., 2021; Cox et al., 2023; Reuter & Spalla King, 2023).

In Ontario, the College of Nurses of Ontario (CNO) is the regulatory body responsible for establishing and maintaining standards for nursing practice and conduct for all nurses (CNO, 2023b). The scope of practice for the Registered Practical Nurse (RPN) role includes providing care and treatment to individuals and families in a variety of healthcare settings such as long-term care homes, hospitals, and community-based settings (CNO, 2023b). Care and treatment provided by RPNs includes assessment, care planning and implementation, medication administration, monitoring and evaluating client care, and health promotion and illness prevention (CNO, 2023a).

The Operating Room Nursing Association of Canada (ORNAC) standards provide guidance to inform the roles and responsibilities of the perioperative nurse, considering such factors as: level of education and experience; the organization's specific policies and procedures that outline the responsibilities of the perioperative nurse in the operating room; client needs (i.e., medical history, diagnosis, and surgical procedure); and staffing needs, as more complex client situations and dynamic clinical environments require RN care (OHA, 2022; ORNAC, 2023). Although there are frameworks to inform staffing decisions, it is not clear how this is operationalized by organizations to inform decisions regarding RPN utilization in the operating room. For example, the ORNAC Position Statement on staffing in the Surgical Suite states that every surgical procedure is staffed by a minimum of two perioperative nursing professionals, one of whom is an RN, and that the primary circulating role shall be assigned to an RN (ORNAC, 2023).

The operating room has been identified by nursing and health care organizations as a practice area for which RPN utilization and scope of practice could be expanded (Chellam Singh & Arulappan, 2023). Some organizations limit RPNs to the scrub role, while other organizations authorize RPNs to practice in both the scrub and circulating roles (Ross, 2006). Within the circulating role, the RPN can be assigned to less complex patients or that of the secondary circulating nurse role (Ross, 2006). Differences in the operationalization of the RPN role within organizations may also be impacted by policies and staffing limitations. For example, if organizational policies and procedures determine that the RPN cannot be the only “circulator,” and the staffing policy for the unit is only two nurses per room, then the RPN role at that organization may be limited to “scrubbing” only.

The current nursing shortage requires further understanding of RPN utilization in the operating room to inform new models of care for perioperative patients (Velji, 2023). RPNs can support the delivery of efficient, high-quality, and effective care; however, various barriers may inhibit RPN utilization in the operating room (Ross, 2006). These barriers may include: lack of recognition for RPN skills and knowledge; limited scope of practice in some jurisdictions; limited education and training to perform all of the competencies required in the operating room; resistance to practice change among health care providers who are accustomed to traditional roles and responsibilities of team members; resource restraints such as limited staffing or funding; and legal and regulatory barriers such as licensure requirements or restrictions on the scope of practice (Ross, 2006). Addressing these barriers through education, professional development, policy changes, and an increased recognition of the value of RPNs can help enhance the utilization of RPNs in the operating room and improve the quality of care for patients (WeRPN, 2020).

The purpose of this study was to explore how RPNs are currently utilized within operating room settings in Ontario, Canada. Three research questions informed the study:

1. How are RPNs utilized within the operating room?
2. What are the facilitators and barriers to RPN utilization in the operating room?
3. What are career development opportunities for RPNs to maximize their utilization within the operating room?

## Methods and Procedures

### Study Design

The study design was a two-phase qualitative descriptive approach. Phase 1 consisted of an online survey. Phase 2 included individual, semi-structured virtual interviews. Interview questions were informed by Phase 1 findings and aimed to further explore participants’ perceptions, attitudes, and beliefs about the facilitators and barriers contributing to RPN utilization in the operating room and career development opportunities.

Ethical approval for the study was obtained from the Toronto Metropolitan University Research Ethics Board (REB# 2023-280) in October 2023. Participation in both phases of the study was voluntary, and participants were informed about their ability to withdraw without penalty prior to providing consent. Informed consent was obtained through an online format, prior to participating in either Phase 1 or Phase 2 of the study.

### Participant Recruitment

The research was conducted in Ontario, Canada. Participants that met the following eligibility requirements were invited to participate: currently working in an operating room [rural, community, or urban hospital and/or private clinic (e.g., private specialty medical treatment clinic, private cosmetic procedure clinic, etc.)] in Ontario, Canada, registered as a nurse (RPN, RN, or NP), and a minimum of one year experience working in the operating room. Phase 1 participants were recruited through two Ontario professional nursing organizations, the College of Nurses of Ontario via a list of CNO members who consented to receive research study participation information by email, and the Registered Practical Nurses Association of Ontario (WeRPN) social media platforms (i.e., Facebook, X, Instagram, and LinkedIn).

### Data Collection and Analyses

The online survey was administered using Google Forms, a survey administration software supported by the university. Potential participants were sent study information by email and accessed the survey link via email through the electronic study recruitment letter or through the WeRPN social media platform study recruitment posts. A follow-up email reminder was sent two weeks after the initial recruitment email for participants to complete the Phase 1 survey (Dillman, 1978). A recruitment reminder was also posted across all WeRPN social media platforms two weeks following the initial social media posts.

Within the survey, participant demographic data was collected including nursing designation, professional role, current work status, gender, highest level of education completed, and city/town of work in Ontario. We also collected responses to seven open-ended descriptive questions related to RPN utilization in the operating room, for example, : “*How are RPNs currently utilized in the operating room in your organization? What are some of the barriers to RPN utilization in the operating room in your organization?”*. Phase 1 survey questions were created and developed by the research team. Deidentified survey data were exported to a Google spreadsheet and stored on a secure shared drive for review and analysis by the research team.

Immediately following the distribution of the survey over online social media platforms, there was a high number of blank responses, suggesting that these responses were potentially fraudulent and possibly generated by online bots attempting to earn the study's incentive. A rigorous algorithm was developed to screen the data for fraudulent responses, an increasing issue in online survey research. Screening methods were used to detect and remove participant responses with incorrect geographical locations, duplicate, not in English, or did not meet other inclusion criteria (Wang et al., 2023).

Participants interested in taking part in a Phase 2 virtual interview indicated their interest following the Phase 1 survey, via a final question that was not linked to their response data. To acknowledge participants’ contribution to the study, a random number draw was used to award five of the Phase 1 online survey participants with a $25 gift card honorarium. In addition, all Phase 2 interview participants received a $25 gift card honorarium to acknowledge their continued participation in the research study. The interviews were conducted using a semi-structured interview guide. Sample interview questions included: “*How have you seen the role of the RPN evolve in the operating room over time? What suggestions do you have for improving RPN utilization in the operating room and RPN career development opportunities*?”. Additional questions addressed facilitators and barriers to RPN utilization in the operating room. Individual interviews were conducted using the secure, university-affiliated Zoom^©^ video and audio-conferencing platform and ranged in length from 30 to 60 min.

Descriptive statistics were used to analyze the participant demographic data for both phases. Qualitative descriptive survey data were de-identified where required, and the research team used a content analysis approach to identify survey codes and categories (Elo & Kyngäs, 2008). The audio-recorded interviews were transcribed verbatim and de-identified. Interview transcripts were read and reread to identify emerging categories from participant responses. The interview transcripts were analyzed inductively using content analysis (Elo & Kyngäs, 2008). All research team members participated in data analysis meetings to review the coding process, review the codebook, and collectively construct codes and categories from the data (Doyle et al., 2020). Emerging codes and categories were compared and reviewed until consensus was achieved.

## Results

### Participant Demographics

A total of 65 Phase 1 online surveys were collected from participants who successfully met the inclusion criteria. Participants included 41 RPNs (63.1%) and 24 RNs (36.9%). Participant professional roles varied by nursing designation. RPN participants were comprised of 38 staff nurses (92.7%), two team leaders (4.9%), and one nurse injector (2.4%). RN participants consisted of 15 staff nurses (62.5%), two team leaders (8.3%), two clinical educators (8.3%), two nurse managers (8.3%), one resource nurse (4.2%), one clinical nurse specialist (4.2%), and one anesthesia assistant (4.2%). Overall, 58 participants (89.2%) identified as female, 6 participants (9.2%) identified as male, and one participant (1.5%) did not disclose their gender identity. Participant levels of education varied by nursing designation, with the majority of RPNs having completed a nursing diploma (63.4%), or a certificate (24.4%), while most RNs completed a baccalaureate degree (58.3%). In addition, some participants completed post-graduate education (e.g., master's degree, doctorate, etc.), including two RPNs (4.8%) and 6 RNs (25.0%). Participants’ years of experience working in the profession ranged from 1 to 37 years with 52 participants (80.0%) indicating they currently work in a hospital setting. Demographic data for Phase 1 participants organized by nursing designation are shown in Table 1.

**Table 1.** Phase 1 Participant Demographic Characteristics.

	RPNs (n = 41)		RNs (n = 24)	
	Frequency	Percentage	Frequency	Percentage
Professional Role				
Staff Nurse (SN)	38	92.7%	15	62.5%
Team Leader (e.g., charge nurse) (TL)	2	4.9%	2	8.3%
Educator in a Practice Setting (EPS)			2	8.3%
Nurse Manager (NM)			2	8.3%
Resource Nurse (e.g., service resource nurse, care coordinator) (RN)			1	4.2%
Clinical Nurse Specialist (CNS)			1	4.2%
RN – Anesthesia Assistant (AA)			1	4.2%
Scrub Assist, Nurse Injector (SA)	1	2.4%		
Current Work Status				
Full-time	26	63.4%	17	70.8%
Part-time	10	24.4%	5	20.8%
Full-time, Part-time	3	7.3%		
Casual	1	2.4%	1	4.2%
Part-time, Casual	1	2.4%	1	4.2%
Gender				
Female	38	92.7%	20	83.3%
Male	2	4.9%	4	16.7%
Did not disclose	1	2.4%		
Highest Level of Education Completed				
Nursing Diploma	26	63.4%	4	16.7%
Baccalaureate Degree	2	4.9%	14	58.3%
Certificate	10	24.4%		
Master's Degree	1	2.4%	5	20.8%
Doctorate	1	2.4%	1	4.2%
Did not disclose	1	2.4%		
Operating Room Training/Specialization				
Yes, perioperative training (e.g., certificate or course)	28	68.3%	17	70.8%
Yes, other (e.g., training in-house, on-the-job training)	9	22.0%	7	29.2%
Yes, but no further disclosure	4	9.8%		
Years Working in the Nursing Profession				
1–5 years	9	22.0%	2	8.3%
6–10 years	8	19.5%	7	29.2%
11–15 years	7	17.1%	2	8.3%
16–20 years	6	14.6%	6	25.0%
21–25 years	2	4.9%	4	16.7%
26–30 years	4	9.8%	1	4.2%
31–35 years	3	7.3%	2	8.3%
36–40 years	2	4.9%		
Years Working in the Operating Room				
1–5 years	18	43.9%	10	41.7%
6–10 years	8	19.5%	2	8.3%
11–15 years	5	12.2%	6	25.0%
16–20 years	2	4.9%	1	4.2%
21–25 years	4	9.8%	3	12.5%
26–30 years	2	4.9%	1	4.2%
31–35 years	2	4.9%	1	4.2%
Work Setting				
Hospital	32	78.1%	20	83.3%
Surgical Clinic within a hospital	3	7.3%	1	4.2%
Free-standing clinic (e.g., endoscopy, cosmetic)	4	9.8%		
Hospital, Surgical Clinic within a hospital	1	2.4%	1	4.2%
Hospital, Free standing clinic (e.g., endoscopy, cosmetic)			2	8.3%
Did not disclose	1	2.4%		
Work Location in Ontario				
Southwest District	12	29.3%	4	16.7%
Lakeland District	7	17.1%	8	33.3%
Eastern District	10	24.4%	1	4.2%
Toronto District	3	7.3%	4	16.7%
Oak Ridges District	4	9.8%	3	12.5%
Western Lake Ontario District	3	7.3%	3	12.5%
Northern District	1	2.4%		
Did not specify city/town (e.g., Ontario)	1	2.4%	1	4.2%

In Phase 2, 13 individual virtual interviews were completed. Eight participants were RPN staff nurses (61.5%), and five were RNs (38.5%) including three staff nurses, one clinical educator, and one nurse manager. Years of experience in the operating room varied from 1 to 26 years of experience, with 69.2% of participants from large urban hospitals.

Following the analysis, survey and interview findings were organized into five categories which reflected factors influencing RPN utilization in the operating room: Specific Healthcare/Practice Setting; Teamwork and Work Culture; Organizational Leadership Practices and Policy Support; Existing Career and Professional Development Opportunities, and the role of Professional Nursing Organizations.

### Specific Healthcare/Practice Setting

Participants described differences in the utilization of RPNs depending on the healthcare setting. For instance, one participant explained that RPNs work to their full scope of practice in an independent healthcare facility setting compared to in a hospital setting: “*I find, though in an independent healthcare facility, you use more of your scope of practice, because there's not as many staff members. […] In a hospital setting, it's a little more difficult to use RPNs because your patients typically have comorbidities […] you have sicker patients, typically it's not quite as cut and clear. But in your private healthcare facility, especially in cosmetic private healthcare facilities, we're not doing surgery on patients that are sick. […] So, a facility such as ours [private], is the perfect spot to utilize RPNs a little bit more to their full extent*.” [Phase 2, P4_RPN].

Another participant noted that in more specialized areas of perioperative medicine, such as endoscopy, there may be more RPN representation: “*where I work now [endoscopy], is mainly run by the RPNs*.” [Phase 2, P8_RN]. One participant suggested hospitals with limited health human resources may rely more heavily on RPNs and as a result, their utilization and scope of practice may be larger: “*I think it kind of depends on the hospital, […] and the more limited resources you have, the bigger scope you have.*” [Phase 2, P11_RN].

RPN participants also described being underutilized in the operating room: *“RPNs may not be given the opportunity to use their full range of skills and knowledge in the operating room, leading to frustration and a sense of underutilization.”* [Phase 1, Q6, 5-RPN-SN]*.* Another participant highlighted: *“RPNs are not utilized to their furthest potential, in fact it is one of the few areas where RPNs are extremely limited in utilizing their full scope of practice and put into the role of scrub nurse only.”* [Phase 1, Q1, 8-RPN-SN]*.*

RPN participants suggested their duties have remained unchanged: *“From the time that I've heard that RPNs have been in the OR, I feel like it hasn't evolved at all. We take on the role of scrub nurse and I feel like that's all we're really allowed to do. They don't even let us document on the computers or anything. […] No charting. Way different than floor nursing, where your responsibilities are so much greater*.” [Phase 2, P10_RPN]*.*

Participants highlighted restrictions in role responsibility and role title. One participant stated, “*My personal experience has been where RPNs are scrub nurses in the operating room.”* [Phase 1, Q1, 7-RN-NM]*.* Further, RPNs were sometimes called “OR techs” and could not engage in extended responsibilities. For example, one participant noted, “*Mostly [RPN's are] in the “scrub” role. For instance, in my workplace, RPN's are referred to as “OR techs” and are not allowed to chart on patients, interview and check in patients, or count with other RPN's.”* [Phase 1, Q2, 28-RPN-SN].

Several participants referred to conflict regarding scope of practice between RPNs and RNs. As one participant noted: *“I feel RPNs could be utilized more efficiently, with policy and procedures put in place and workplace culture change. We have been perceived as a threat to our coworkers, the RNs, and there has been conflict regarding the scope of practice. When we first took [on]-call, we were told it was unsafe. Now, it's scheduled.”* [Phase 1, Q1, 9-RPN-SN]*.* Another participant commented *“There are people, doctors and nurses, that don't believe that we have the scope of practice or the critical thinking skills that allow us to be able to work in an operating room.”* [Phase 2, P9_RPN]*.* Despite their essential role in participating in surgical safety checklists, one participant expressed that RPNs often feel overlooked stating: *“Even though we do our safety checklists at the beginning, the RPNs are the forgotten people.”* [Phase 2, P6_RPN].

Participants commented on the traditional roles and utilization of the RN and RPN within the operating room setting, and how these role traditions are beginning to be challenged: *“I think in the operating room, with the older set, there [are] well-steeped traditions that aren't crossed. And even with some RPNs at my facility, they're not willing to go outside of their regular role. I think there's a barrier in attitude is really what it is for both parties. The younger groups now, those attitudes are changing, those lines are “blurred”. We don't see it as a class difference like it used to be.”* [Phase 2, P11_RN].

### Teamwork and Work Culture

Participants described a culture of teamwork with team members including nursing (RNs and RPNs), anesthesia and surgery. Participants acknowledged the value of working together with nursing colleagues from different backgrounds, designations, and experiences. As one participant remarked: “*You get a multi-disciplinary team of nurses […] you're bringing different levels of nursing and bringing different people with different experiences to the team. So, whenever you do that, you get multiple perspectives on what should be done and what's the proper outcome for the patient.*” [Phase 2, P5_RPN]. One RN participant highlighted the expertise that RPNs bring to the operating room and their proficiency in scrubbing: “*So really utilizing [RPNs] because they know what is immediately needed, or what's important, to open, because they're right there at the bedside.*” [Phase 2, P8_RN]. Another RN participant reflected an appreciation of the RPN role in the operating room*: “[…] an RPN is just as valuable as an RN, just different responsibilities. But like I said, it just depends on the experience of the RN and the RPN. I’ve worked with wonderful RPNs that just have worked in the OR long enough with their experience that they can point things out to me that I can learn from. They're really skilled with the equipment. Having an RPN versus. an RN, the difference is negligible, for sure.”* [Phase 2, P11_RN]*.*

Other participants spoke about the operating room work environment culture and more specifically senior nurses in the work environment who are opposed to change. One participant stated*: “[…] some of the senior nurses who are just so averse to change. And these are the nurses who actually contributed to a lot of the toxicity in our OR environment.”* [Phase 2, P1_RN]. The reluctance to change the responsibilities of RPNs in the operating room was also noted by another participant: *“People making up their own rules. They think that this is the way it is, and that's the way it's always been. And so that's what needs to happen.”* [Phase 2, P4_RPN].

Further, some participants described tensions of fear and apprehension regarding changing scopes of practice among RNs and RPNs. “*Given that RPNs will be introduced later this year in our hospital, there's a lot of fear and anger amongst the perioperative RNs.”* [Phase 1, Q6, 32-RN-SN]*.* Another participant highlighted the apprehension experienced by some RNs when asked about their thoughts on increasing the scope of practice of the RPN in the operating room: “*[…] there were concerns noted that, okay, so if [RPNs] expand to circulating, why do you need RNs? Why would you pay more money for an RN? […] [The] RN role is feeling a bit threatened in the OR […] they worry about their job.”* [Phase 2, P13_EPS]*.*

Other participants described the stigma that sometimes comes with the RPN title as such: *“I feel like, there's a little bit of a stigma that comes with the RPN title in the OR with those older personalities.”* [Phase 2, P3_NM]. A survey participant also added “*Occasionally, some of my RN coworkers seem to look down on RPNs – say comments like you are just an RPN – but mostly my RN coworkers appreciate the skill and expertise RPNs bring to the OR as it takes years to become a proficient scrub nurse*” [Phase 1, Q1, 2-RPN-SN]*.*

### Organizational Leadership Practices and Policy Support

Some participants spoke to the benefits of having supportive management and leadership in their workplace, as one participant described: “*My manager was very “gung-ho” about RPNs broadening their scope and did a lot of work with the hospital to convince them that it was appropriate, and that we had the RPNs that could do it.”* [Phase 2, P9-RPN]. Another participant described the benefits of having supportive management, managers that “*[…] back you up, and that actually want you to progress and do better […] and once management is advocating for the RPNs, then there would be more cooperation, because my coworkers, my RN coworkers, I feel for the most part, are supportive. Especially the newer RNs*.*”* [Phase 2, P10-RPN]. However, others suggested leadership and management beliefs were barriers to the increased utilization of RPNs. One survey participant identified: “*Lack of knowledge or understanding to our scope, leadership is not interested in changing status quo.*” [Phase 1, Q6, 2-RPN-SN].

Within the specific context of the organization or healthcare facility policies and organizational beliefs were highlighted as factors influencing RPN utilization in the operating room. As one participant noted: “*Politics. The hospital system does not allow RPNs to go beyond their basic scope of practice, regardless of advanced training.*” [Phase 1, Q6, 22-RPN-SN]*.* While another participant noted that some hospitals may be more supportive of RNs in comparison to RPNs: “*Hospital is very pro-RN”* [Phase 1, Q6, 62-RPN-SN]*.*

Another participant noted that the absence of a formal job description, contributed to limited opportunities for professional growth and recognition for RPNs’ overtime: *“When I first started full-time in the OR 14 years ago […] we were introduced without any job, description, policy, or procedure, or patient standard of care. So, we were not treated the best. We were not given any encouragement or empowerment. So you are to scrub, and that's it. And basically, that's all. They have never progressed any further.”* [Phase 2, P7_RPN].

### Existing Career and Professional Development Opportunities

Participants described various career and professional development opportunities that support increased utilization of RPNs in the operating room, such as mentorship programs and educational resources. One participant expressed the following: *“Mentorship: Many hospitals offer mentorship programs for new RPNs, where they are paired with an experienced RPN who can provide guidance and support. Educational resources: Hospitals often have resources such as simulation labs, where RPNs can practice their skills in a safe environment. Many hospitals also offer continuing education opportunities, such as online courses and workshops.”* [Phase 1, Q4, 3-RPN-SN].

Further, participants highlighted the need for more RPNs in leadership positions in their workplaces, to advocate for RPNs and specifically for their increased utilization in the operating room. As one participant explained: *“I really believe if we had an RPN in a middle management, non-management supervisory role, I think that would help.”* [Phase 2, P6_RPN]. Some participants identified that there are career development opportunities for experienced RPNs to train to second circulate, and to take on a clinical or service lead role. Another participant also identified the opportunity for RPNs to become a service lead in settings such as cystoscopy but also acknowledged the lack of leadership opportunities available for RPNs: *“I know that the RPNs did have the opportunity to become a service lead, because mainly, for example, like cystoscopy, it's more like a day-to-day kind of patient, there's no anesthesia. So, they're able to just kind of run that area on their own. And they had the opportunity, if they wanted to, to apply for a team lead or service lead position. But other than that, I am not aware of, and I know I've heard some [RPNs] say that they wish they had more opportunities. But what kind of opportunities are out there, I'm not too sure.”* [Phase 2, P8_RN].

For RPNs keen on expanding their scope of practice, one participant suggested they consider demonstrating their willingness to invest and grow in their career: *“My suggestion for the RPNs in the OR is to consider […] the other roles because they are likely going to move forward with training them in the circulating role. […] Start to take little initiatives to see how things work from the RN circulating role perspective, to show kind of an interest and investment in growing into those roles as well*. [Phase 2, P13_EPS]*.*

Other participants acknowledged the lack of further career and professional development available for RPNs in Ontario and expressed that there are limited opportunities to further one's nursing career without pursuing an undergraduate nursing degree to first become an RN. One participant highlighted the following: “*Currently, being an RPN, or an LPN for that matter, across Canada, there are virtually no programs that certify you, that allow you to go further than that. The [OR] certificate is one of the furthest things that we can do as an RPN and it's unfortunate because we can be utilized to a lot more capacity than that, especially those that have a lot of experience.”* [Phase 2, P5_RPN].

Another participant remarked that when they were in the process of bridging from RPN to RN, their ten years of experience as an RPN was deemed insufficient and they were required to take an RN perioperative nursing course in order to satisfy the anesthesia competency requirement: *“I do think that education for RPNs in the OR, […] there is a difference in the programs. There is an RN perioperative nursing program, and there's an RPN perioperative nursing program. So, it focuses on, I think, scrubbing for the RPN, and the RN kind of does both, with the anesthesia component. So, I went to a college outside of [City in Ontario], […] and I had to prove when I got hired as an RN, that I did the anesthesia component for them to hire me as an RN, even though I had 10 years of RPN experience in the OR.”* [Phase 2, P3-NM].

Participants advocated that further education should be available for RPNs interested in broadening their scope of practice to include competencies such as circulating, assisting in anesthesia and intubation, and in administering medications. As one participant endorsed: “*Open our scope to circulate and First Assist with further education! We are capable!”* [Phase 1, Q5, 40-RPN-SA]. Another participant spoke to the capabilities of RPNs and that they are more than capable in broadening their scope of practice in the operating room: “*RPNs are more than capable of assisting anesthesia in the intubation process, if we could have further training to understand pushing medications. I am currently ACLS certified and if RPNs could all have the opportunity to take this course, we could broaden the scope and broaden the utilization of RPNs in the OR.”* [Phase 1, Q5, 41-RPN-SN].

### Role of Professional Nursing Organizations

Participants described the roles of professional associations and unions in influencing RPN utilization in the operating room. For example, one participant remarked that the recent changes to the scope of practice guidelines from the College of Nurses of Ontario (CNO) have encouraged more flexibility in the RPN and RN roles, and will allow for expanded utilization of RPNs in the operating room: “ *[…] the scope of practice with CNO has actually changed quite a bit in the last addition, […] it's not as prescriptive as to what an RPN and RN can do. It's more based on knowledge, skill, and judgment. I think because of that, you're seeing a lot of changes on different units. Whereby they have a bit more flexibility as to what the RPN role can do and cannot do so the OR is sort of looking at that as well.”* [Phase 2, P13_EPS]*.*

While participants recognized the RPN union as an example of support that currently exists to facilitate RPN utilization in the operating room, some participants also acknowledged the opportunities for the RPN union. As one participant explained: “*[RPNs are] at the bottom of the totem pole because we're a different union. Even if I've had 14 years seniority at the hospital, my seniority is there within the hospital, but within the OR I'm at the bottom. […] So new grads that come in […] they have 2 months of nursing under their belts, have more seniority than me in the OR, and they'll get offered a promotion before me. So that's disheartening.”* [Phase 2, P10_RPN]. Another participant added that one solution would be to make the RPN union a nursing union: “*Our union could be a nursing union and not have us lumped into non nursing unions.”* [Phase 1, Q5, 46-RPN-SN].

## Discussion

In a recent mixed methods study, Weiser et al. (2024) explored the barriers and facilitators to integrating RPNs in acute care hospitals, and found that having RPNs practice to full scope is complex and multifactorial. Similar to Weiser et al. (2024), our findings indicate multiple factors influencing the utilization of RPNs in the operating room. The specific healthcare/practice setting can influence the degree to which RPNs practice to full scope. For example, participants suggested that RPNs working in independent healthcare organizations worked to full scope, whereas RPNs in hospitals did not necessarily have the same opportunity. Participants further suggested that expanding the RPN scope of practice in the operating room would support greater perceived efficiency and more productive use of nursing resources. Chellam Singh and Arulappan (2023) have suggested operating room leadership often has a focus on cost reduction through supporting efficiencies in care and therefore further enhancing patient access to healthcare services.

Our study findings further elaborated on the disconnect between how RPNs are currently utilized, versus how RPNs want to be utilized in the operating room and how they hope to progress in their careers. Weiser et al. (2024) described the importance of RPNs as critical members of the nursing and interprofessional team noting the importance of ensuring RPNs feel valued within their roles in acute care settings.

In our study, RPNs expressed the need for more opportunities to take on positions of leadership in their organizations. While the CNO's Entry-to-Practice Competencies for RPNs (revised June 2023) include a statement referencing leadership for RPNs, “*demonstrate leadership, direction and supervision to unregulated health workers and others*”, further opportunity for suggesting how RPNs can move forward could be considered for future positions in leadership (CNO, 2023a). The CNO and other professional nursing and operating room associations across Ontario and Canada could consider outlining the steps that RPNs and healthcare organizations can take to increase the opportunity for experienced RPNs to take on positions of leadership in the operating room.

In this study, teamwork and work culture were described as having an important impact on utilization of RPNs in the operating room. Weiser et al. (2024) found similar results in their exploration of utilizing RPNs across broader acute care units. For example, they found that team dynamics, RN/RPN conflict, fear of changing scopes of practice, or pre-existing beliefs about RPN roles from RNs may create potential challenges for integrating RPNs in acute care settings. The tension between RNs and RPNs role and responsibilities was evident in our study and that of Weiser et al. (2024), who suggest the importance of engaging in discussions and staff education regarding roles, responsibilities as a critical component prior to integrating RPNs into new acute care practice settings.

Our study findings have implications for a variety of health system organizations, across the domains of clinical practice, education, and policy. For middle and senior leaders in healthcare organizations, our findings could support policy and practice changes that address nursing shortages in the operating room by adjusting RPNs scope of practice to support increased utilization (Lankshear & Martin, 2019). Managers could foster communication about the unit workload and how all members of the healthcare team could support one another (Nowrouzi-Kia et al., 2022). Formal planning and education with organizational leaders, frontline RNs and RPNs, and interprofessional team members, pre-integration of RPNs, is important to facilitate RPN integration in acute care settings (Weiser et al., 2024). The additional utilization of RPNs in the operating room can offer leaders more flexibility in staffing and care delivery models. As the RPN role in the operating room continues to grow and evolve over time, professional nursing and operating room associations in Ontario and across Canada can continue to evaluate and revise their professional nursing standards of practice to reflect growing RPN practice competencies.

In terms of career and professional development opportunities for RPNs, our findings identify gaps and highlight opportunities to support targeted education for post-licensure RPNs in the operating room. Continuing education programs within post-secondary institutions, healthcare organizations offering site specific RPN education and training or professional organizations can support ongoing development of RPNs not only for the clinical skills required in the operating room but also for leadership roles within specific surgical areas.

### Limitations and Implications for Future Research

This study explored the utilization of RPNs in the operating room within the province of Ontario. Future research could explore RPN utilization across other jurisdictions to gain a broader understanding of this phenomena. In the recruitment of participants for the Phase 1 survey, selection bias could have occurred as only CNO members who consented to receive research information by email were sent the study participation information. To reach a broader audience and circumvent this selection bias, we also recruited participants through WeRPN's social media platforms. Given this was a qualitative study with a small number of participants, findings are not generalizable to the greater population of operating room nurses across Ontario, as those who participate do not necessarily represent the larger population of RPNs. However, we did recruit from a range of geographic regions across the province. Further, we had research team members with perioperative experience involved in the research process, which supported a rigorous and contextually rich analysis and interpretation of the findings.

## Conclusion

With the ongoing health human resources challenges, healthcare organizations continue to explore and re-evaluate the roles and contributions of RPNs across various settings. Given the complex nature of healthcare systems, new models of care, and scopes of practice for all healthcare providers continue to evolve. It is therefore critical to consider how RPNs can be utilized to support perioperative care. The findings from this study advance discussions of how reconceptualizing the RPN role in specialty settings, such as the operating room, can ease current staffing shortages, productively challenge traditional models of care and enhance career/professional development opportunities for RPNs. The growing body of evidence on barriers and facilitators to implementing RPN roles in practice provides actionable ways forward to strengthen the nursing workforce through role optimization – It is time to put the evidence into action.

## Supplemental Material

sj-docx-1-cjn-10.1177_08445621251345337 - Supplemental material for Optimizing the Role of Registered Practical Nurses in the Operating Room: A Two-Phase Qualitative Descriptive Study Supplemental material, sj-docx-1-cjn-10.1177_08445621251345337 for Optimizing the Role of Registered Practical Nurses in the Operating Room: A Two-Phase Qualitative Descriptive Study by Sherry Espin, Sue Bookey-Bassett, Alyssa Indar, Victoria Pringle, Don Rose, Elaine Santa Mina and Juliette Teodoro in Canadian Journal of Nursing Research

## References

[bibr1-08445621251345337] Ball K. Doyle D. Oocumma N. I. (2015). Nursing shortages in the OR: Solutions for new models of education. AORN Journal, 101(1), 115–136. 10.1016/j.aorn.2014.03.015 25537332

[bibr2-08445621251345337] Brand S. L. Thompson Coon J. Fleming L. E. Carroll L. Bethel A. Wyatt K. (2017). Whole-system approaches to improving the health and wellbeing of healthcare workers: A systematic review. PloS One, 12(12), e0188418. 10.1371/journal.pone.0188418 PMC5714334 29200422

[bibr3-08445621251345337] Brooks C. Della Ratta C. LaSala M. E. Gerberich B. Browne K. (2021). Leveraging academic-clinical partnerships to create an operating room nurse pipeline. JONA: The Journal of Nursing Administration, 51(3), 168–172. 10.1097/NNA.0000000000000989 33570375

[bibr4-08445621251345337] Chellam Singh B. Arulappan J. (2023). Operating room nurses’ understanding of their roles and responsibilities for patient care and safety measures in intraoperative practice. SAGE Open Nursing, 9. 10.1177/23779608231186247 PMC10350747 37465651

[bibr5-08445621251345337] College of Nurses of Ontario. (2023a). Entry-to-practice competencies for registered practical nurses. CNO. https://www.cno.org/globalassets/docs/reg/41042_entrypracrpn-2020.pdf

[bibr6-08445621251345337] College of Nurses of Ontario. (2023b). Practice standard: Scope of practice. CNO. https://cno.org/Assets/CNO/Documents/Standard-and-Learning/Practice-Standards/49041-scope-of-practice.pdf

[bibr7-08445621251345337] Cox C. W. Rumble M. M. McNelis A. M. Walsh H. A. (2023). An examination of the career choices of nursing graduates after completing a perioperative elective. AORN Journal, 117(4), e1–e9. 10.1002/aorn.13895 36971529

[bibr8-08445621251345337] Dillman D. A. (1978). Mail and telephone surveys: The total design method (Vol. 19, p. 375). Wiley.

[bibr9-08445621251345337] Doyle L. McCabe C. Keogh B. Brady A. McCann M. (2020). An overview of the qualitative descriptive design within nursing research. Journal of Research in Nursing, 25(5), 443–455. 10.1177/1744987119880234 34394658 PMC7932381

[bibr10-08445621251345337] Elo S. Kyngäs H. (2008). The qualitative content analysis process. Journal of Advanced Nursing, 62(1), 107–115. 10.1111/j.1365-2648.2007.04569.x 18352969

[bibr11-08445621251345337] Lankshear S. Martin D. (2019). Getting comfortable with “it depends”: Embracing the impermanence of scope of practice. Nursing Leadership (1910-622X), 32(1). 10.12927/cjnl.2019.25850 31228343

[bibr12-08445621251345337] Nowrouzi-Kia B. Fox M. T. Sidani S. Dahlke S. Tregunno D. (2022). The comparison of role conflict among registered nurses and registered practical nurses working in acute care hospitals in Ontario Canada. Canadian Journal of Nursing Research, 54(2), 112–120. 10.1177/08445621211014421 PMC9109589 34042538

[bibr13-08445621251345337] Ontario Hospital Association. (2022). Practical solutions to maximize health human resources. OHA. https://www.nosm.ca/wp-content/uploads/2022/04/OHA-Practical-Solutions-to-Maximize-HHR.pdf

[bibr14-08445621251345337] Operating Room Nurses Association of Canada. (2023). ORNAC standards, guidelines, and position statements for perioperative registered nurses. ORNAC. https://www.ornac.ca/en/standards

[bibr15-08445621251345337] Registered Practical Nurses Association of Ontario. (2020). Ontario RPN survey. WeRPN. https://www.werpn.com/wp-content/uploads/2021/10/WeRPN-2020-Survey-FINAL-1.pdf

[bibr16-08445621251345337] Reuter J. Spalla King T. (2023). A perioperative specialty elective to address the perioperative nursing shortage: Student perspectives and outcomes. AORN Journal, 117(6), 376–383. 10.1002/aorn.13931 37235621

[bibr17-08445621251345337] Ross J. (2006). Extending the perioperative circulating role for the registered practical nurse. WeRPN. https://www.werpn.com/wp-content/uploads/2019/12/Extending-the-Perioperative-Circulating-Role.pdf

[bibr18-08445621251345337] Rozario S. Y. Sarkar M. Farlie M. K. Lazarus M. D. (2024). Responding to the healthcare workforce shortage: A scoping review exploring anatomical pathologists’ professional identities over time. Anatomical Sciences Education, 17(2), 351–365. 10.1002/ase.2260 36748328

[bibr19-08445621251345337] Sutton C. Prowse J. McVey L. Elshehaly M. Neagu D. Montague J. Alvarado N. Tissiman C. O’Connell K. Eyers E. Faisal M. Randell R. (2023). Strategic workforce planning in health and social care–an international perspective: A scoping review. Health Policy, 132, 104827. 10.1016/j.healthpol.2023.104827 37099856

[bibr20-08445621251345337] Velji K. (2023). Innovating health care – transforming the role of nurses. Canadian Nurse. https://www.canadian-nurse.com/blogs/cn-content/2023/05/01/transforming-the-role-of-nurses

[bibr21-08445621251345337] Wang J. Calderon G. Hager E. R. Edwards L. V. Berry A. A. Liu Y. Dinh J. Summers A. C. Connor K. A. Collins M. E. Prichett L. Marshall B. R. Johnson S. B. (2023). Identifying and preventing fraudulent responses in online public health surveys: Lessons learned during the COVID-19 pandemic. PLOS Global Public Health, 3(8), e0001452. 10.1371/journal.pgph.0001452 PMC10446196 37610999

[bibr22-08445621251345337] Weiser N. Dissanayake M. Santiago C. Harrington F. Benny Gerard N. Dimmock S. Canzian S. Topolovec-Vranic J. (2024). The registered practical nurse (RPN) role in an academic acute care hospital: A mixed method study of the barriers and facilitators to practice. Journal of Nursing Management, 2024(1), 7309242. 10.1155/2024/7309242 40224844 PMC11919085

