# Supplementary figures and images for: Optimizing the Role of Registered Practical Nurses in the Operating Room: A Two-Phase Qualitative Descriptive Study
Source: Can J Nurs Res. 2025 Jun 3;57(4):497–506. doi: 10.1177/08445621251345337 (PMC12589658; doi:10.1177/08445621251345337)

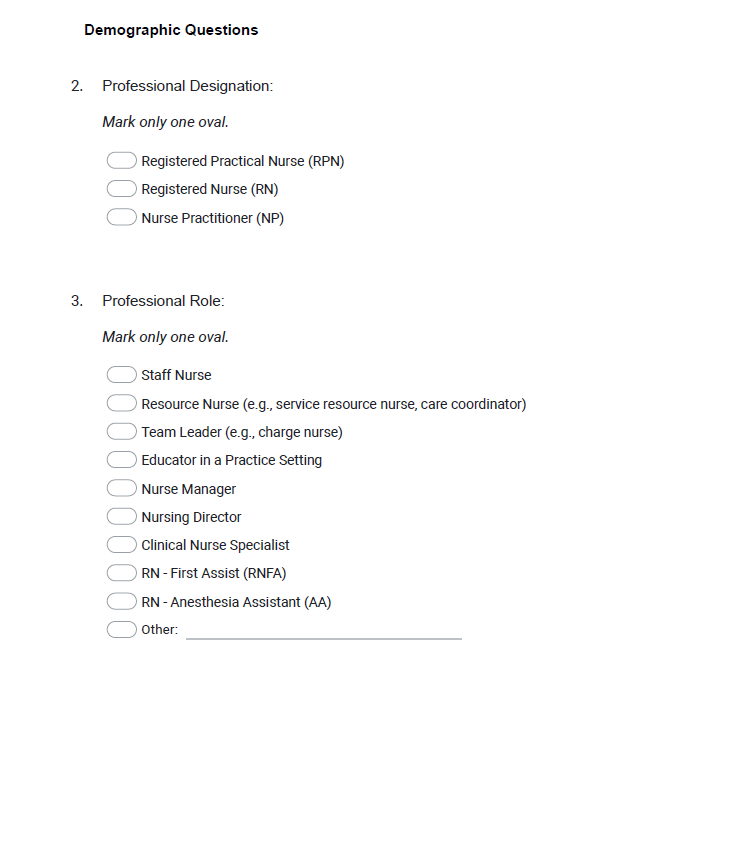


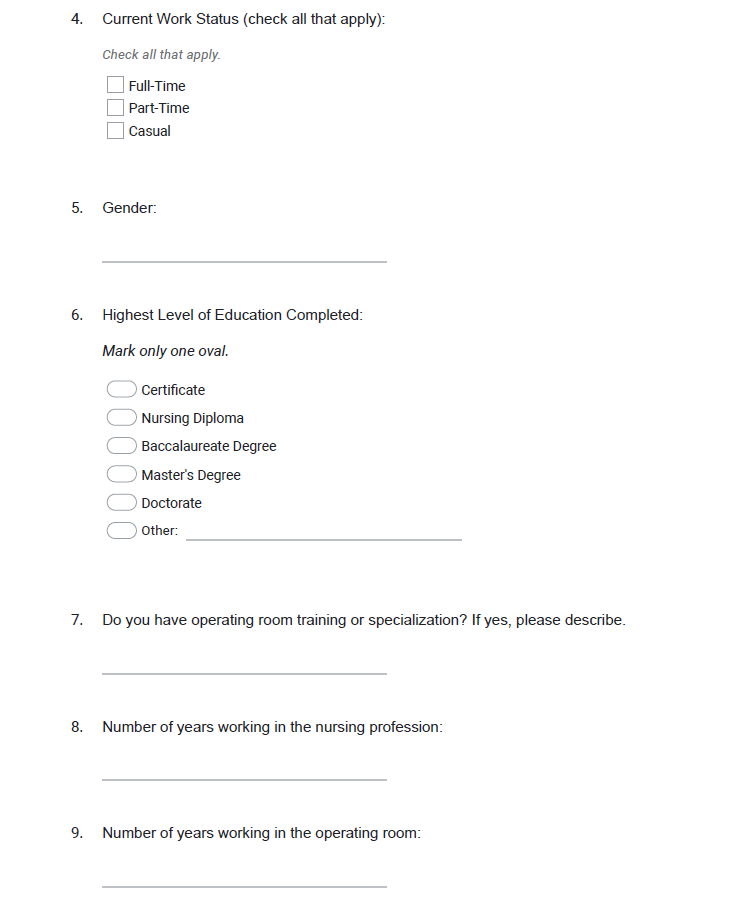


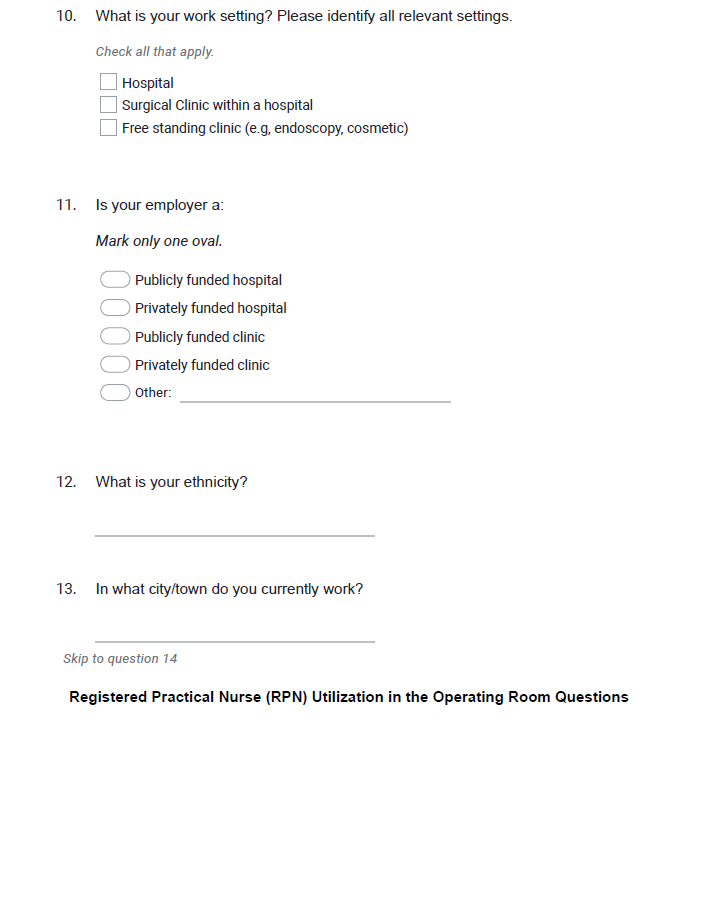


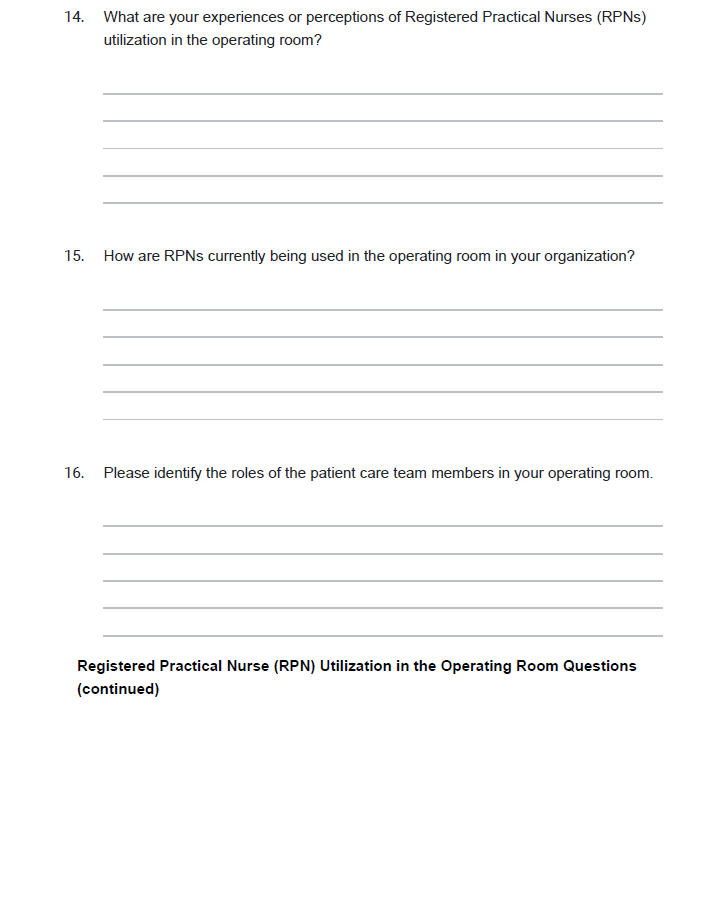


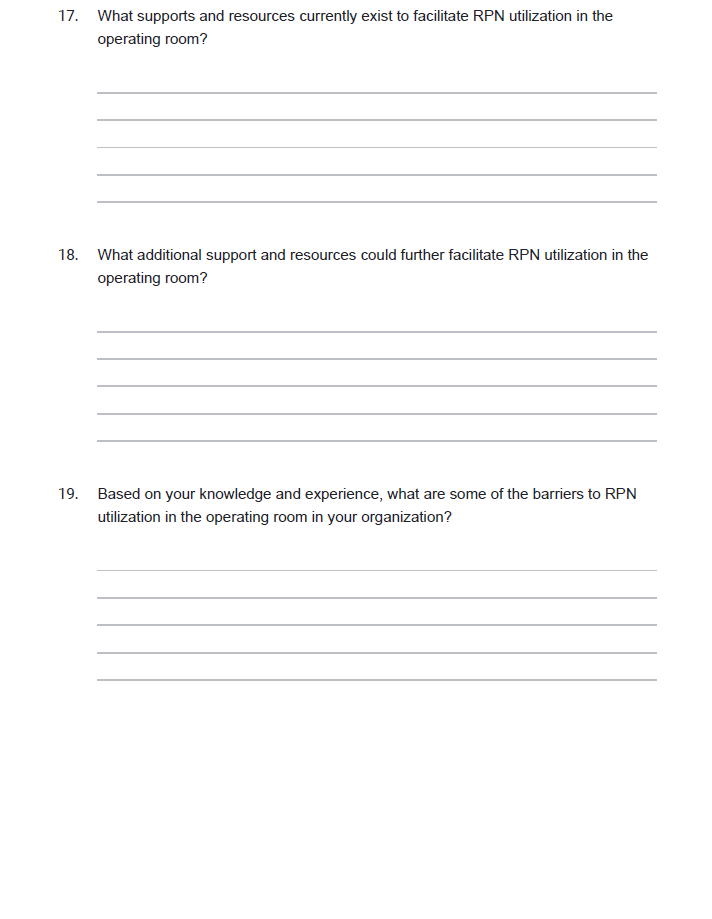


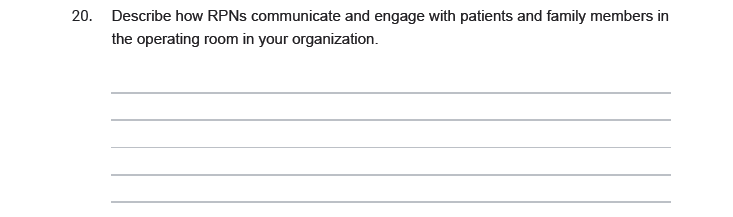

Supplement: sj-docx-1-cjn-10.1177_08445621251345337 - Supplemental material for Optimizing the Role of Registered Practical Nurses in the Operating Room: A Two-Phase Qualitative Descriptive Study [file sj-docx-1-cjn-10.1177_08445621251345337.docx]
